# An analysis of first-time enquirers to the CancerBACUP information service: variations with cancer site, demographic status and geographical location

**DOI:** 10.1038/sj.bjc.6690023

**Published:** 1999-09-24

**Authors:** M Boudioni, K McPherson, J Mossman, M Boulton, A L Jones, J King, E Wilson, M L Slevin

**Affiliations:** CancerBACUP, 3 Bath Place, Rivington Street, London, EC2A 3DR, UK; Department of Epidemiology and Population Health, London School of Hygiene and Tropical Medicine, Keppel Street, London, WC1E 7HT, UK; Department of Epidemiology and Public Health, Imperial College School of Medicine, St Mary's Hospital, Norfolk Place, London, W2 1PG, UK; Royal Free Hospital, Pond Street, London, NW3 2QG, UK; Cancer Research Campaign, 10 Cambridge Terrace, London, NW1 JL, UK; Department of Health, Wellington House, 135–155 Waterloo Road, London, SE1 8UG, UK; Department of Oncology, St Bartholomew's Hospital, West Smithfield, London, EC1A 7BE, UK

**Keywords:** cancer information, demographic data, population data, standardized incidence ratios

## Abstract

A retrospective comparison of cancer incidence data and, where relevant, population data with 16 955first-time users (patients, relatives and friends) of a national cancer information service (CancerBACUP) during the period April1995 to March 1996 is presented. The number of events observed was compared with the number of events expected, were the nationalrates of cancer incidence and population demographics apply. Standardized incidence ratios (SIRs) (observed – expectedratios) were used to indicate any differences. Statistically significant differences (*P*< 0.001) in the observed andexpected sex, age and primary site distribution of patients enquired about were found. Statistically significant differences(*P*< 0.001) were also identified for the age, employment status, socioeconomic class and geographical location offirst-time enquirers (patients, relatives and friends). Enquiries about brain, testis and breast cancers and non-Hodgkin'slymphoma (NHL) were substantially higher than expected; enquiries about bladder, lung, stomach and colorectal cancers were muchlower than expected. As the service is provided via a freephone number, it is available to all, and users might be expected to berandomly distributed across the variables listed. The underlying reasons for the differences identified need to be investigated,and the role of information in the care of cancer patients should be formally evaluated. © 1999 Cancer Research Campaign


*British Journal of Cancer* (1999) **79**(1), 138–145
© 1999 Cancer Research Campaign

### An analysis of first-time enquirers to the CancerBACUP information service: variations with cancer site, demographic status and geographical location

**M Boudioni[1], K McPherson[2], J Mossman[1], M Boulton[3], AL Jones[4], J King[5], E Wilson[6] and ML Slevin[7]**

[1]CancerBACUP, 3 Bath Place, Rivington Street, London EC2A 3DR; [2]Department of Epidemiology and Population Health, London School of Hygiene and Tropical Medicine, Keppel Street, London WC1E 7HT; [3]Department of Epidemiology and Public Health, Imperial College School of Medicine, St Mary's Hospital, Norfolk Place, London W2 1PG; [4]Royal Free Hospital, Pond Street, London NW3 2QG; [5]Cancer Research Campaign, 10 Cambridge Terrace, London NW1 4JL; [6]Department of Health, Wellington House, 135–155 Waterloo Road, London SE1 8UG; [7]Department of Oncology, St Bartholomew's Hospital, King George V Building, West Smithfield, London EC1A 7BE, UK

**Summary** A retrospective comparison of cancer incidence data and, where relevant, population data with 16 955 first-time users (patients, relatives and friends) of a national cancer information service (CancerBACUP) during the period April 1995 to March 1996 is presented. The number of events observed was compared with the number of events expected, were the national rates of cancer incidence and population demographics apply. Standardized incidence ratios (SIRs) (observed – expected ratios) were used to indicate any differences. Statistically significant differences ($P < 0.001$) in the observed and expected sex, age and primary site distribution of patients enquired about were found. Statistically significant differences ($P < 0.001$) were also identified for the age, employment status, socioeconomic class and geographical location of first-time enquirers (patients, relatives and friends). Enquiries about brain, testis and breast cancers and non-Hodgkin's lymphoma (NHL) were substantially higher than expected; enquiries about bladder, lung, stomach and colorectal cancers were much lower than expected. As the service is provided via a freephone number, it is available to all, and users might be expected to be randomly distributed across the variables listed. The underlying reasons for the differences identified need to be investigated, and the role of information in the care of cancer patients should be formally evaluated.

**Keywords:** cancer information; demographic data; population data; standardized incidence ratios

The need for information and support for cancer patients is well documented (Audit Commission, 1993). Patients contacting cancer information services in the USA request information on the disease itself, the treatment (including treatment options) and mechanisms for coping (Meissner et al, 1990; Manfredi et al, 1993). Patients participating in a clinical trial overwhelmingly (94%) expressed a desire for as much information as possible from their oncologist, including information about the disease, all treatment options, treatment side-effects and the chance of cure (Fallowfield et al, 1994, 1995). Significant others (categorized as friends and relatives in this study) requested similar information (Meissner et al, 1990). A large percentage of family members feel that their needs are not adequately met by health care providers (Houts et al, 1991). It has been suggested that the family understanding, acceptance and participation in the patient's care is a determining factor in the effectiveness of the treatment plan (Conatser, 1986; Hardwick and Lawson, 1995).

The Calman–Hine Report recommended: 'patients, families and carers should be given clear information and assistance in a form they can understand about treatment options and outcomes available to them at all stages of treatment from diagnosis onwards' (Expert Advisory Group, 1995). Many patients reported to CancerBACUP that they did not receive any information when they were given their original diagnosis, but this may reflect the fact that a patient who is anxious and overwhelmed has more trouble processing and recollecting information (Harris, 1998). As recently as November 1997, East Dorset Community Health Council reported: 'Given the amount of information available from a number of different sources it was disappointing that nearly half the patients interviewed were of the view that they had not been given the information and support they required or indeed that they had been offered any at all!' (East Dorset Community Health Council, 1997). Patients do express the view when asked that it is important to receive full information in order to avoid confusion, uncertainty, fear and anxiety (Fallowfield et al; 1994, 1995; Meredith et al, 1996; National Cancer Alliance, 1996). Others have the view that limited information is appropriate to their needs or admit that they cannot absorb any information initially because they are too traumatized (Manfredi et al, 1993; National Cancer Alliance, 1996).

BACUP (now called CancerBACUP) was established in 1984 by the late Dr Vicky Clement-Jones as a result of her own experience of cancer. She recognized that information helped patients and their carers to understand how the disease and treatment might affect them, to anticipate problems and to plan their lives accordingly (Clement-Jones, 1985). The charity provides a national service giving information, emotional support, counselling and practical advice to cancer patients, their families and friends. Specialist cancer nurses staff a telephone information service, they receive ongoing training in communication and counselling skills and are kept abreast of current practice by attending training

*Received 5 December 1998*
*Revised 14 April 1998*
*Accepted 17 May 1998*

*Correspondence to:* M Boudioni

**Table 1** Major cancer sites by sex of patient. Observed CancerBACUP first-time enquiry rates (from patients, relatives, friends) in April 95/March 96, expected CancerBACUP enquiry rates if Great Britain 1991 cancer incidence rates apply and standardized incidence ratios (SIRs) for major cancer sites by sex of patient

| Site description | Male patients | | Female patients | | All patients | |
|---|---|---|---|---|---|---|
| | No. | 95% CI | No. | 95% CI | No. | 95% CI |
| Female breast | | | | | | |
| Observed | | | 4570 | | 4570 | |
| Expected | | | 2735 | | | |
| SIR | | | 1.67 | (1.62–1.72) | 1.67 | (1.62–1.72) |
| Lung | | | | | | |
| Observed | 935 | | 555 | | 1490 | |
| Expected | 1534 | | 1065 | | 2599 | |
| SIR | 0.61 | (0.57–0.65) | 0.52 | (0.48–0.57) | 0.57 | (0.54–0.60) |
| Colorectal | | | | | | |
| Observed | 760 | | 605 | | 1365 | |
| Expected | 852 | | 1237 | | 2089 | |
| SIR | 0.89 | (0.83–0.97) | 0.49 | (0.45–0.53) | 0.65 | (0.62–0.69) |
| Prostate | | | | | | |
| Observed | 980 | | | | 980 | |
| Expected | 854 | | | | 854 | |
| SIR | 1.15 | (1.08–1.22) | | | 1.15 | (1.08–1.22) |
| Non-Hodgkin's lymphoma | | | | | | |
| Observed | 510 | | 385 | | 895 | |
| Expected | 216 | | 272 | | 488 | |
| SIR | 2.36 | (2.16–2.58) | 1.42 | (1.28–1.56) | 1.83 | (1.72–1.96) |
| Ovary | | | | | | |
| Observed | | | 760 | | 760 | |
| Expected | | | 478 | | 478 | |
| SIR | | | 1.59 | (1.48–1.71) | 1.59 | (1.43–1.71) |
| Leukaemia (all) | | | | | | |
| Observed | 355 | | 235 | | 590 | |
| Expected | 178 | | 202 | | 380 | |
| SIR | 1.99 | (1.79–2.21) | 1.16 | (1.02–1.32) | 1.55 | (1.43–1.68) |
| Brain | | | | | | |
| Observed | 365 | | 190 | | 555 | |
| Expected | 109 | | 115 | | 224 | |
| SIR | 3.35 | (3.01–3.71) | 1.65 | (1.43–1.91) | 2.48 | (2.28–2.69) |
| Stomach | | | | | | |
| Observed | 255 | | 160 | | 415 | |
| Expected | 378 | | 348 | | 726 | |
| SIR | 0.67 | (0.59–0.76) | 0.46 | (0.39–0.54) | 0.57 | (0.52–0.63) |
| Bladder | | | | | | |
| Observed | 255 | | 120 | | 375 | |
| Expected | 510 | | 282 | | 792 | |
| SIR | 0.50 | (0.44–0.57) | 0.43 | (0.35–0.51) | 0.47 | (0.43–0.52) |
| Oesophagus | | | | | | |
| Observed | 260 | | 105 | | 365 | |
| Expected | 192 | | 204 | | 396 | |
| SIR | 1.35 | (1.19–1.53) | 0.51 | (0.42–0.62) | 0.92 | (0.83–1.02) |
| Cervix | | | | | | |
| Observed | | | 305 | | 305 | |
| Expected | | | 344 | | 344 | |
| SIR | | | 0.89 | (0.79–0.99) | 0.89 | (0.79–0.99) |
| Testis | | | | | | |
| Observed | 215 | | | | 215 | |
| Expected | 84 | | | | 84 | |
| SIR | 2.56 | (2.23–2.93) | | | 2.56 | (2.23–2.93) |
| Lip and mouth (all) | | | | | | |
| Observed | 75 | | 95 | | 170 | |
| Expected | 138 | | 107 | | 245 | |
| SIR | 0.54 | (0.43–0.68) | 0.89 | (0.72–1.09) | 0.69 | (0.59–0.81) |
| Other | | | | | | |
| Observed | 1455 | | 1585 | | 3040 | |
| Expected | 1375 | | 2281 | | 3656 | |
| SIR | 1.05 | (1.00–1.11) | 0.70 | (0.66–0.73) | 0.83 | (0.80–0.86) |
| All known malignant neoplasms (unknown-sex patients, general and unknown-cancer enquiries are excluded) | | | | | | |
| Observed | 6420 | | 9670 | | 16090 | |
| Expected | 6420 | | 9670 | | 16090 | |
| Chi-square | 375.42 $P < 0.001$ | | 505.71 $P < 0.001$ | | 768.54 $P < 0.001$ | |

Ninety-five per cent confidence intervals for the SIRs are given in parentheses.

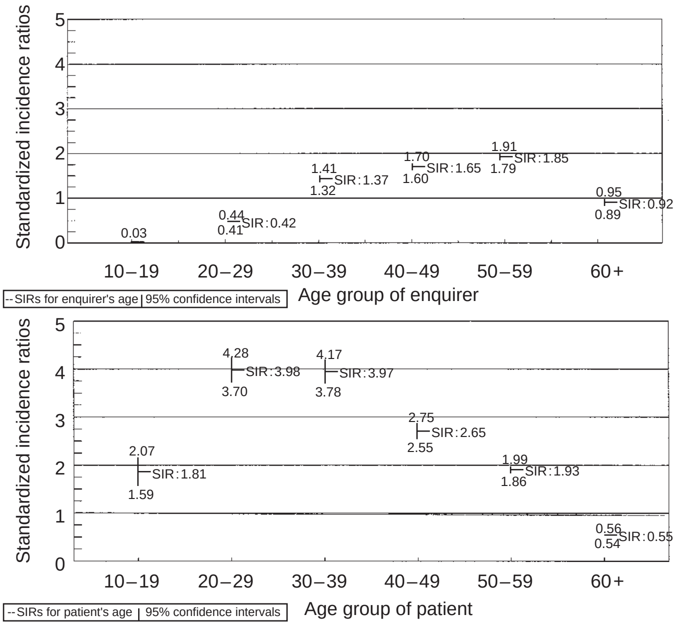

**Figure 1** Standardized incidence ratios (SIRs) for age of enquirer and age of patient enquired about courses and scientific meetings, reviewing the literature and by lectures from external speakers. From the outset, data about users of the service have been collected (Slevin et al, 1988).

A number of organizations provide independent information and support to cancer patients, their relatives and friends. They also help to fill the gap between the patients' (and carers') needs and what is provided by the Health Service. In view of the numbers of newly diagnosed patients in the UK, it is clear that not all patients and carers access the services available. There is little evidence to support a view that patients choose not to seek independent sources of information and support, but many CancerBACUP users indicate a lack of knowledge about other sources of help. There has been little systematic detailed evaluation of the characteristics of users of a national cancer information service. This paper examines whether the population using CancerBACUP Information Service (CIS) for the first time is representative of the population of Great Britain; and whether the patients enquired about are representative of the population who develop cancer. This is a first step in examining the role of independent information in cancer care.

### MATERIALS AND METHODS

An 'enquirer record form' is completed for each fifth enquirer of CancerBACUP CIS; information is recorded about the enquirer, the patient (if different from the enquirer), the disease, demographic details, type of request(s) and advice given. Ethnic group data were collected from August 1996 onwards, after the study period. Disclosure of information is voluntary and not complete for all enquirers. If the enquirer is distressed the nurse does not ask any question considered inappropriate. Details of missing data are provided at the specific sections in the Results. An extensive coding system is used to classify details of the enquiry including a maximum of six subjects of enquiry and five codes for advice given. The forms are checked thoroughly and coded before being entered on to the database. Information collected during the first 2 years of CancerBACUP (then named BACUP) was reported previously (Slevin et al, 1988). Demand for the service outstrips capacity, particularly following media activity on new treatments. Up to six lines were open at any time during the study period; the number of calls diverted to an answering machine was recorded by a call-logging machine, but the number of callers obtaining an engaged signal (when all the lines, including the answering machine, are busy) is unknown.

Data from first-time enquirers in the categories of patients, relatives and friends, during the period 1 April 1995 to 31 March 1996, were compared with the distribution of the population of Great Britain in 1991 (Office of Population, 1993; Office of Population, 1994). Data on patients enquired about from first-time enquirers were compared with the distribution of cancer incidence in Great Britain in 1991 (ISD, National Health Service in Scotland, 1996; Office for National Statistics, 1996). Enquiries originating from outside England, Scotland and Wales are excluded. It was assumed that relatives and friends were resident in the same health authority as the patient. Age, sex, site-specific tumour type, socioeconomic status and health authority of residence were compared. Observed to expected ratios for the above items are presented as standardized incidence ratios (SIRs) with 95% confidence intervals. Statistical significance was calculated using the chi-square method. Analyses were done separately for England, Wales and Scotland and pooled for presentation.

### RESULTS

All cancers have been included in the study with the exception of non-melanoma skin cancer, which is under-registered (Cancer Research Campaign, 1994; Office for National Statistics, 1996). There were 212 000 registrations of malignant neoplasms in England and Wales in 1991 (1990 for Wales): 104 000 (49.1%) in males and 108 000 (50.9%) in females (Office for National Statistics, 1996). In Scotland, there were 23 690 registrations in

 *© Cancer Research Campaign 1999*

**Table 2** Median ages of patients enquired about from CancerBACUP first-time enquirers in April 95/March 96 and median ages of patients, Great Britain 1991 cancer incidence

| | CancerBACUP patients ($n$ = 15 475) | | GB cancer incidence ($n$ = 235 759) | | Median age difference (cancer incidence–CancerBACUP) |
|---|---|---|---|---|---|
| **Site description** | **No.** | **Median age[a]** | **No.** | **Median age** | |
| Female breast | 4495 | 52.0 | 33 785 | 62.9 | 10.9 |
| Lung | 1420 | 67.1 | 41 299 | 71.0 | 3.9 |
| Colorectal | 1305 | 60.8 | 30 830 | 72.7 | 11.9 |
| Prostate | 945 | 71.7 | 15 277 | 75.8 | 4.1 |
| Non-Hodgkin's lymphoma | 860 | 56.0 | 7195 | 67.7 | 11.7 |
| Ovary | 720 | 57.9 | 5858 | 65.4 | 7.5 |
| Leukaemia (all) | 570 | 55.4 | 5644 | 69.9 | 14.5 |
| Brain | 520 | 50.1 | 3453 | 58.6 | 8.5 |
| Stomach | 405 | 61.7 | 11 160 | 74.1 | 12.4 |
| Bladder | 375 | 70.3 | 12 717 | 72.4 | 2.1 |
| Oesophagus | 355 | 67.3 | 6000 | 72.5 | 5.2 |
| Cervix | 290 | 40.8 | 4230 | 52.0 | 11.2 |
| Testis | 215 | 30.9 | 1517 | 40.5 | 9.6 |
| Lip and mouth (all) | 165 | 56.4 | 3848 | 66.4 | 10.0 |
| All known malignant neoplasms (unknown age patients are excluded) | 15 475 | 56.6 | 235 759 | 70.1 | 13.5 |

[a]The highest age coding group at CancerBACUP in April 95–March 96 was 60+. Therefore it has been assumed that there is a linear age distribution for the 60+ age group and two-thirds of those patients were aged 60–75 years. Prostate has been excluded from this assumption.

**Table 3** Observed employment status of CancerBACUP first-time enquirers (patients, relatives, friends) in April 95/March 96, expected employment status if Great Britain 1991 population rates apply and standardized incidence ratios (SIRs) for employment status

| | CancerBACUP first-time enquirers ($n$ = 16 070) | | | |
|---|---|---|---|---|
| **Employment status** | **Observed** | **Expected** | **SIR** | **95% CI** |
| Economically active | | | | |
| Employed | 10 515 | 8900 | 1.18 | (1.16–1.20) |
| Unemployed[a] | 605 | 1569 | 0.39 | (0.36–0.42) |
| Economically inactive | | | | |
| Students | 295 | 614 | 0.48 | (0.43–0.54) |
| Retired | 2500 | 3046 | 0.82 | (0.79–0.85) |
| Other inactive (housepersons) | 2155 | 1941 | 1.11 | (1.06–1.16) |
| Total population aged 16+ (unknown employment status enquirers are excluded) | 16 070 | 16 070 | | |
| Chi-square = 234.51, $P < 0.001$ | | | | |

[a]There was not a separate coding category for 'permanently sick' enquirers at CancerBACUP. They were coded as 'unemployed'. Therefore, the two GB census categories 'unemployed' and 'permanently sick' have been combined for the comparison. Ninety-five per cent confidence intervals for the SIRs are given in parentheses.

the same year; 11 474 (48.4%) in males and 12 216 (51.6%) in females (ISD, National Health Service in Scotland, 1996).

During the study period, 38 765 enquiries were answered by the Cancer Information Service. Of these, health professionals, students, the 'worried well' and others made 7660 enquiries. The majority of enquirers (31 105; 80% of all 38 765 enquirers) represented diagnosed patients (13 955, 36%) and relatives and friends of patients (17 150, 44%). When asked whether they had used any of the CancerBACUP services previously, 11 930 (38.3%) of the 31 105 patients, relatives and friends replied that they had used at least one of the services (publications, information, counselling) previously and 2220 (7.1%) were unclear or did not clarify; these two groups are excluded. The study population comprised 16 955 (54.5%) patients, relatives and friends who replied that they had not used any of the services. This sample represents 8% of new cancer cases in Great Britain in 1991. As previously described, not every item was recorded for each caller; items not recorded were coded 'unknown' and have been excluded from the analysis.

### Sex and cancer site

The sex of enquirer was known for all 16 955 first-time enquirers. There was an excess of female first enquirers (77.7%) compared with the proportion of women in the Great Britain population (51.5%) and fewer male enquirers (22.3%) compared with the proportion of men (48.5%) in the Great Britain population in 1991

© Cancer Research Campaign 1999

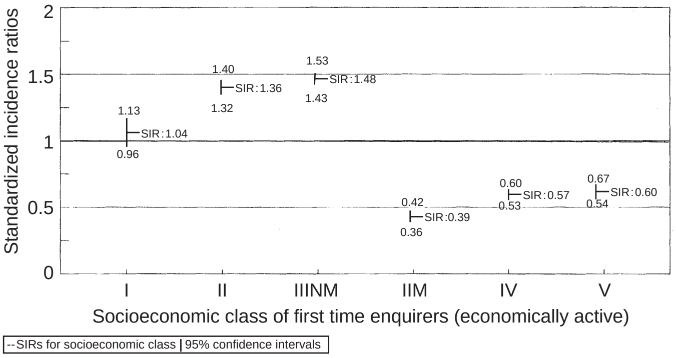

**Figure 2** Standardized incidence ratios (SIRs) for socioeconomic class of enquirers

(*P* < 0.001). The SIRs are, respectively, 1.51 (CI 1.48–1.53) for females and 0.46 (CI 0.45–0.48) for males.

The sex of patient enquired about was known for the vast majority of the first-time enquiries (16 875, 99.5%). The enquiry rates for male and female patients are closer to the cancer incidence rates, but there is still an excess of calls about women with cancer (60.1%) compared with the incidence of cancer in women (50.9%) (*P* < 0.001). The number of enquiries about men with cancer is lower than expected if cancer incidence rates in 1991 apply (39.9% vs 49.1%, *P* < 0.001). The SIRs are 1.18 (CI 1.16–1.20) for females and 0.81 (CI 0.79–0.83) for males.

There were 16 090 (94.9%) first-time enquiries from patients, relatives and friends where the sex of patient and tumour site were known (Table 1). For both sexes combined, the most common cancer sites were breast, lung, colorectal and prostate, accounting for 52.2% of all first-time enquiries. For male patients, prostate, lung and colorectal were the most common sites for enquiry, not reflecting the frequency of 1991 cancer registrations, which were lung, prostate and colorectal in that order. For female patients the most common cancer registrations were breast, colorectal and lung in that order (ISD, National Health Service in Scotland, 1996; Office for National Statistics, 1996), whereas CancerBACUP enquiries were mostly about breast, ovary and colorectal cancer.

When enquiry rates are compared with incidence rates for specific cancers (Table 1), a number of differences emerge. For both male and female patients, the enquiry rate is higher than expected for brain tumours (SIR = 2.48), non-Hodgkin's lymphoma (SIR = 1.83) and leukaemia (SIR = 1.55). In contrast, enquiry rates are lower than expected for bladder (SIR = 0.47), stomach (SIR = 0.57) and lung (SIR = 0.57). Enquiry rates for females are higher than expected for breast (SIR = 1.67) and ovary (SIR = 1.59), whereas for males they are much higher for testis cancer (SIR = 2.56). Interestingly, for prostate and cervix cancers, the number of enquiries is closer to the expected values (SIR for prostate = 1.15; SIR for cervix = 0.89). Enquiry rates for oesophageal cancer are noteworthy for the substantial difference between the female ratio (SIR = 0.51) and the male ratio (SIR = 1.35).

### Age

The age of enquirer was known for 16 365 enquirers (96.5% of first-time enquiries). Compared with the population, the enquiry rate from the study population (patients, relatives, friends) aged below 30 years is less than expected (SIRs < 0.45) and for ages 30–60 is greater than expected (SIRs > 1.35), the peak being from

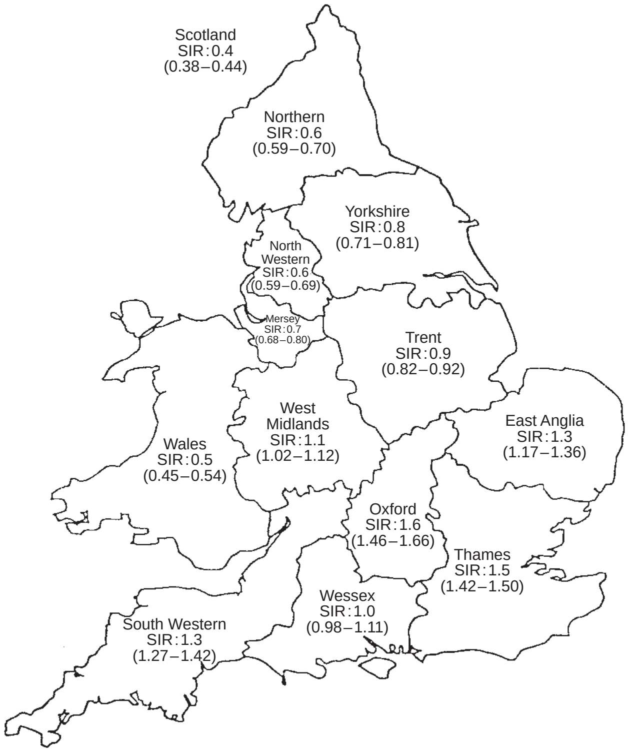

**Figure 3** Standardized incidence ratios (SIRs) for health authorities of residence of enquirers. Ninety-five per cent confidence intervals are given in parentheses those aged 50–59 (SIR = 1.85) (Figure 1). It is interesting to note that the age distribution of users residing in England and Wales differs from that seen in Scotland; in England and Wales the highest SIR is noted in the 50–59 age group (SIR = 1.87), while in Scotland the highest SIR is noted in the 30–39 age group (SIR = 1.86).

For specific cancer enquiries, the age of patient enquired about by the study population was known for 15 475 patients (91.3% of first-time enquiries). (It is not known how many of these calls were from multiple family members or friends about the same patient.) For patients aged under 60 years, the enquiry rate was much higher than expected (Figure 1), especially for the age range 20–39 years, but lower than expected for patients aged over 60 years (SIR = 0.55). A further breakdown by age for patients over 60 is not possible as the data were collected at the time for '60 years and over'.

*© Cancer Research Campaign 1999*

The overall median age for patients enquired about was 56.6 years, which is 13.5 years less than the median age for cancer of 70.1 years (Table 2). For each cancer site individually, the median age of patients enquired about was lower than the incidence age.

### Employment status and socioeconomic class

The employment status was known for 16 070 enquirers (94.8% of first-time enquirers). The enquiry rate from employed people and housepersons is close to but exceeds the population rate (Table 3). However, the enquiry rate for the unemployed (SIR = 0.39), students (SIR = 0.48) and retired people (SIR = 0.82) is less or significantly less than expected if Great Britain population rates in 1991 apply (Office of Population, 1994).

There were 10 515 economically active enquirers; almost all of them (10 510) could be classified in one of the socioeconomic classes according to their occupation (Office of Population, 1990*a*). There is no actual difference in observed and expected rates in socioeconomic class I, if Great Britain population rates in 1991 apply (Figure 2). However, there are higher enquiry rates from the other non-manual classes and significantly lower rates of enquiry from the manual classes.

### Health authority of residence

The health authority of residence was coded for 16 025 enquirers (94.5% of first-time enquirers). As previously explained, it was assumed that relatives and friends resided in the same health authority as the patient did. Fewer than expected enquiries from Scotland (SIR = 0.4) and Wales (SIR = 0.5) are recorded if cancer incidence rates in 1991 apply (Figure 3). Although England as a whole receives approximately the number of expected enquiries (SIR = 1.10), the SIRs for the regional health authority of residence differ significantly. Higher enquiry rates than expected (SIRs range = 1.0 to 1.6) are observed for the south and central parts of England. The northern parts (Northern, Yorkshire, Trent, Mersey and North Western) present lower enquiry rates than expected (SIRs range = 0.6–0.9).

### DISCUSSION

CancerBACUP is the largest independent provider of cancer information in the UK, answering 38 765 enquiries from England, Scotland and Wales during the period 1 April 1995 to 31 March 1996. Slightly more than half of the patients, relatives and friends categories are first-time users, and their age, sex, social class, etc distribution might, a priori, be expected to reflect the Great Britain population. Similarly, the age, sex and primary site distribution of patients enquired about for the first time might be expected to reflect the cancer incidence. There are, however, important differences between the distribution of Great Britain population and CancerBACUP users, and between the distribution of cancer registration and patients enquired about. These differences have not changed substantially since the analysis of the first 30 000 users reported that ‘users were predominantly middle class, between the ages of 30 and 49 and living in south-east England’ (Slevin et al, 1988), although in the current study the highest SIR for enquirers’ age was for the 50–59 age group.

Manfredi et al (1993) found that information non-seekers in the USA are more likely to be male and over 60 years of age. The population in the present study had an excess of female enquirers and female patients enquired about. This may reflect significant gender differences in the use and utilization of social support (Greenglass, 1992; Harrison et al, 1995). Harrison et al (1995) found that male cancer patients are much more likely to have used only one confidante in time of crisis, usually their partner, while women made use of a wider circle of family, friends and partner and used more confidantes overall. Health and social support behaviour practised by men could influence their use of information services. The age of patients enquired about was substantially lower than the age of newly diagnosed cancer patients. Slevin et al (1988) speculated that the younger age of service users reflected the greater impact of a cancer diagnosis in early life in a society where longevity is expected.

Unemployed people present significantly low enquiry rates (Table 3). Manual workers were, in 1988, and still are, under-represented in users (Figure 2). Kogevinas (Office of Population, 1990*b*), demonstrating class differences in terms of cancer incidence and survival in a longitudinal study, suggested that people from lower social classes make less effective use of health services. Other studies have shown that the degree to which a cancer patient seeks information depends on his or her educational, cultural and financial background (Harris, 1998). Low-literacy individuals are less likely to seek information (Manfredi et al, 1993) and low literacy is more prevalent among individuals of low socioeconomic status (Brown et al, 1993). Anecdotally, clinicians have expressed the view that patients from lower socioeconomic classes are less likely to question their doctor’s views and treatment decisions, making additional information less relevant.

There are noticeable differences between the number of enquiries relating to specific cancers (Table 1). Some are over-represented, such as NHL, leukaemia and testicular cancer, whereas others are under-represented, such as bladder and lung cancers. This may relate to the younger age distribution of patients enquired about and the fact that the over-represented cancers have lower median age of diagnosis (Figure 1 and Table 2). It is not possible to confirm or refute this proposition since age at diagnosis was not collected for the study population. There could be other possible reasons for these differences. For example, the commonly held view is that little can be done about lung cancer, and this may not encourage the seeking of further information or support. A number of survivors from testicular cancer have been frank about their experiences in the media, and this may have an impact. Such issues need to be examined.

In 1988, the predominance of calls about breast cancer was observed as ‘striking’ (Slevin et al, 1988). During the current study period, despite the increased availability of specialist breast cancer nurses and breast cancer organizations, breast cancer remains responsible for an excess of calls compared with the expected number (SIR = 1.67; Table 1). Media activity is likely to be a key determinant of information seeking and breast cancer is frequently the subject of media reports. A study of 210 cancer patients and carers found that 100% sought information from sources outside the health care team, 38% from media sources (Shingler et al, 1997). During the study period 12.5% of all CancerBACUP enquirers found out about the organization from media (unpublished data).

Several studies have shown that patients with cancer generally felt poorly informed about their disease, although the requirements for information vary from individual to individual (Martin et al, 1992; Manfredi et al, 1993; Fallowfield et al, 1994). The needs of carers also vary and are dynamic throughout the cancer experience (Hardwick and Lawson, 1995). There is limited information about the provision of information for different cancers and subgroups of

© Cancer Research Campaign 1999

the population. Manfredi et al (1993) saw no significant differences in the information-seeking behaviour of cancer patients according to their cancer site, although the study only covered five sites: colon, breast, lung, lymphoma and prostate.

Differences in the geographical locations of users compared with the population distribution may reflect differences between rural and urban areas and between socioeconomic groups. Inequalities in health and NHS resource allocation exist across UK regions, although there is unlikely to be a simple relationship between these factors and the cancer incidence and survival rates (Hart, 1985; Stationary Office, 1998). Levels of urban health seem to be generally worse than in rural areas (Watt et al, 1994). Health care services accessibility is a central problem and, though not uniformly experienced, rural populations have poorer access than others (Watt et al, 1994). White et al (1996) found that knowledge deficit proved to be one of the most frequently identified problems of cancer patients in rural areas. If regional differences reflect unmet need rather than different needs, steps should be taken to address the issue. The National Cancer Alliance (1996) found that patients expressed most of the same needs in four different areas, but there were some clear differences in how these needs were being met. It reported that patients would like written information to be actively given to them by the health care team. Incorporating information provision within the service agreements for cancer would be a major step forward to ensuring that information provision becomes more evenly distributed. The Clinical Outcomes Group has produced guidance for purchasers on breast and colorectal cancers, which includes specific recommendations for the provision of information (Cancer Guidance sub-group of the Clinical Outcomes Group, 1996, 1997). However, no additional funding has been provided, and it is not clear how this might be achieved.

CancerBACUP has recommended that everyone affected by a diagnosis of cancer should have access to a range of information and emotional and social support tailored to their own particular needs (BACUP, 1996). Information for, and support to, people with cancer will often reduce uncertainty and might improve the quality of life for many (Ley, 1976; Audit Commission, 1993; Fallowfield et al, 1995; The National Cancer Alliance, 1996; White et al, 1996). It is interesting to speculate that the provision of information to women with breast cancer – if information plays a role in compliance with or benefit from treatment (Conatser, 1986) – may have contributed to the recently demonstrated improved survival from breast cancer (Beral et al, 1995). The effectiveness of information in improving the quality of lives of cancer patients, their relatives and friends needs to be better understood.

This analysis shows that the population using a national cancer information service for the first time does not reflect the general population and the cancer patients enquired about do not reflect the population that develops cancer. There is a clear need to identify the reasons why some patients do – and some do not – seek help from independent organizations, not least to clarify if this results from a lack of knowledge about the availability of such help. The situation has changed little since Meredith et al (1995) reported that ‘basic research into patients’ needs for information which remain unfulfilled by interaction with doctors and nurses is urgently needed’.

### ACKNOWLEDGEMENTS

This study was supported by the Cancer Research Campaign. We thank all the nurses who worked in CancerBACUP Information Service during the period under study and those that helped with the data analysis. We thank Sandra Monks for her patience while typing the manuscript.

### REFERENCES

Audit Commission (1993) *What Seems to be the Matter: Communication between Hospitals and Patients*. (NHS Report No 12) HMSO: London

BACUP (1996) *The Right to Know*: *A BACUP Guide to Information and Support for People Living with Cancer*. BACUP: London

Beral V, Hermon C, Reeves G and Peto R (1995) Sudden fall in breast cancer death rates in England and Wales. *Lancet* **345**: 1642–1643

Brown P, Ames N, Mettger W, Smith TJ, Gramarossa GL, Friedell GH and McDonald SS (1993) Closing the comprehension gap: low literacy and the cancer information service. Monographs, *J Natl Cancer Inst* **14**: 157–164

Expert Advisory Group on cancer to the chief medical officers of England and Wales (1995) *A Policy Framework for Commissioning Cancer Services*. Department of Health: London

Cancer Guidance sub-group of the Clinical Outcomes Group (1996) *Good Practice Guidance for Purchasers*: *Improving Outcomes in Breast Cancer – The Manual*. NHS Executive: Leeds

Cancer Guidance sub-group of the Clinical Outcomes Group (1997) *Good Practice Guidance on Commissioning Cancer Services*: *Improving Outcomes in Colorectal Cancer – The Manual*. NHS Executive: London

Cancer Research Campaign (1994) *Incidence – UK; Cancer Research Campaign Factsheet*. Cancer Research Campaign: London

Clement-Jones V (1985) Cancer and beyond: the formation of BACUP. *Br Med J* **291**: 1021–1023

Conatser C (1986) Preparing the family for their responsibilities during treatment. *Cancer* **58**: 508–511

East Dorset Community Health Council (1997) *Experiences of Cancer Services. Patients and Carers’ Perspectives in East Dorset*. East Dorset Community Health Council: Bournemouth

Fallowfield L, Ford S and Lewis S (1994) Information preferences of patients with cancer. *Lancet* **344**: 1576

Fallowfield L, Ford S and Lewis S (1995) No news is not good news: information preferences of patients with cancer. *Psycho-Oncology* **4**: 197–202

Greenglass ER (1992) A gender-role perspective of social support: implications for psychological functioning. *Int J Psychol* **27**: 601

Hardwick C and Lawson N (1995) The information and learning needs of the caregiving family of the adult patient with cancer. *Eur J Cancer Care* **4**: 118–121

Harris K (1998) The information needs of patients with cancer and their families. *Cancer Pract* **6(1)**: 39–46

Harrison J, Maguire P and Pitceathly C (1995) Confiding in crisis: gender differences in pattern of confiding among cancer patients. *Soc Sci Med* **41**: 1255–1260

Hart N (1985) Human health and society. In *The Sociology of Health and Medicine*, Haralambos M (ed.), pp. 55–62. Causeway Press: Lancashire

Houts PS, Rusenas I, Simmonds MA and Hufford DL (1991) Information needs of families of cancer patients: a literature review and recommendations. *J Cancer Ed* **6/4**: 255–261

ISD, National Health Service in Scotland (1996) *Incidence and Prevalence of All Malignant Neoplasms in Scotland*. ISD Unpublished data: Edinburgh

Ley P (1976) Towards better doctor–patient communications. In *Communication Between Doctors and Patients*, Jennet AE (ed.), pp. 77–98. Oxford University Press: Oxford

Manfredi C, Czaja R, Price J, Buis M and Janiszewski R (1993) Cancer patients’ search for information. Monographs, *J Natl Cancer Inst* **14**: 93–104

Martin A, Leone L, Castagliuolo I, Di Mario F and Naccarato R (1992) What do patients want to know about their inflammatory bowel disease? *Ital J Gastroenterol* **24**: 477–480

Meissner HI, Anderson DM and Odenkirchen JC (1990) Meeting information needs of significant others: use of the cancer information service. *Pat Ed Couns* **15**: 171–179

Meredith C, Symonds P, Webster L, Lamont D, Pyper E, Gillis CR and Fallowfield L (1996) Information needs of cancer patients in west Scotland: cross sectional survey of patients’ views. *Br Med J* **313**: 724–726

Meredith P, Emberton M and Wood C (1995) New directions in information for patients. *Br Med J* **311**: 4–5

National Cancer Alliance (1996) *“Patient-Centered Cancer Services”? What Patients Say*. National Cancer Alliance: Oxford

 *© Cancer Research Campaign 1999*

Office for National Statistics (1996) Monitor, Population and Health, MB1 96/1 In *Registrations of Cancer Diagnosed in 1991, England and Wales*. Office for National Statistics: London

Office of Population, Censuses and Surveys (1990*a*), Employment Department Group *Standard Occupational Classification*. HMSO: London

Office of Population, Censuses and Surveys (1990*b*), *1971–1983 Longitudinal Study*: *Socio-Demographic Differences in Cancer Survival*. Kogevinas E (ed.) HMSO: London

Office of Population, Censuses and Surveys (1993) *1991 Census, Sex, Age and Marital Status, Great Britain*. HMSO: London

Office of Population, Censuses and Surveys (1994) *Topic Monitor*; *1991 Census, Economic Activity, Great Britain*. Office of Population, Censuses and Surveys: London

Shingler G, Balusu R and Thomas R (1997) Where do patients seek additional information after a diagnosis of cancer – a multicentre survey? Paper presented at the European Cancer Conference, Hamburg. *Eur J Cancer* **33 (S8)**: S321

Slevin ML, Terry Y, Hallet N, Jefferies S, Launder S, Plant R, Wax H and McElwain T (1988) BACUP – the first two years: evaluation of a national cancer information service. *Br Med J* **297**: 669–672

Stationary Office (1998) *Our Healthier Nation, A Contract for Health* (A Consultation Paper) Stationary Office: London

Watt IS, Franks AJ and Sheldon TA (1994) Health and health care of rural populations in the UK: is it better or worse? *J Epidemiol Commun Health* **48**: 16–21

White NJ, Given BA and Devoss DN (1996) The advanced practice nurse: meeting the information needs of the rural cancer patient. *J Cancer Ed* **11**: 203–209

*© Cancer Research Campaign 1999*

## References

[PDF_00660] Beral V., Hermon C., Reeves G., Peto R. (1995). Sudden fall in breast cancer death rates in England and Wales.. Lancet.

[PDF_00662] Brown P., Ames N., Mettger W., Smith T. J., Gramarossa G. L., Friedell G. H., McDonald S. S. (1993). Closing the comprehension gap: low literacy and the Cancer Information Service.. J Natl Cancer Inst Monogr.

[PDF_00676] Clement-Jones V. (1985). Cancer and beyond: the formation of BACUP.. Br Med J (Clin Res Ed).

[PDF_00678] Conatser C. (1986). Preparing the family for their responsibilities during treatment.. Cancer.

[PDF_00683] Fallowfield L., Ford S., Lewis S. (1994). Information preferences of patients with cancer.. Lancet.

[PDF_00685] Fallowfield Lesley, Ford Sarah, Lewis Shon (1995). No news is not good news: information preferences of patients with cancer.. Psychooncology.

[PDF_00689] Hardwick C., Lawson N. (1995). The information and learning needs of the caregiving family of the adult patient with cancer.. Eur J Cancer Care (Engl).

[PDF_00692] Harris K. A. (1998). The informational needs of patients with cancer and their families.. Cancer Pract.

[PDF_00694] Harrison J., Maguire P., Pitceathly C. (1995). Confiding in crisis: gender differences in pattern of confiding among cancer patients.. Soc Sci Med.

[PDF_00699] Houts P. S., Rusenas I., Simmonds M. A., Hufford D. L. (1991). Information needs of families of cancer patients: a literature review and recommendations.. J Cancer Educ.

[PDF_00707] Manfredi C., Czaja R., Price J., Buis M., Janiszewski R. (1993). Cancer patients' search for information.. J Natl Cancer Inst Monogr.

[PDF_00709] Martin A., Leone L., Castagliuolo I., Di Mario F., Naccarato R. (1992). What do patients want to know about their inflammatory bowel disease?. Ital J Gastroenterol.

[PDF_00712] Meissner H. I., Anderson D. M., Odenkirchen J. C. (1990). Meeting information needs of significant others: use of the Cancer Information Service.. Patient Educ Couns.

[PDF_00715] Meredith C., Symonds P., Webster L., Lamont D., Pyper E., Gillis C. R., Fallowfield L. (1996). Information needs of cancer patients in west Scotland: cross sectional survey of patients' views.. BMJ.

[PDF_00718] Meredith P., Emberton M., Wood C. (1995). New directions in information for patients.. BMJ.

[PDF_00742] Slevin M. L., Terry Y., Hallett N., Jefferies S., Launder S., Plant R., Wax H., McElwain T. (1988). BACUP--the first two years: evaluation of a national cancer information service.. BMJ.

[PDF_00747] Watt I. S., Franks A. J., Sheldon T. A. (1994). Health and health care of rural populations in the UK: is it better or worse?. J Epidemiol Community Health.

[PDF_00750] White N. J., Given B. A., Devoss D. N. (1996). The advanced practice nurse: meeting the information needs of the rural cancer patient.. J Cancer Educ.

